# Integrating Oral Health Care into Primary Health Care System

**DOI:** 10.5402/2012/657068

**Published:** 2012-02-29

**Authors:** Abolghasem Hajizamani, Tayebeh Malek Mohammadi, Ebadollah Hajmohammadi, Shahin Shafiee

**Affiliations:** ^1^Dental Public Health Department, Kerman Dental School, Kerman University of Medical Sciences, Iran; ^2^Dental Public Health Department, Kerman Oral and Dental Disease Research Center, Kerman Dental School, Kerman University of Medical Sciences, Kerman, Iran; ^3^Kerman Dental School, Kerman University of Medical Sciences, Kerman, Iran; ^4^Community Medicine Department, Kerman Central Health Center, Kerman, Iran

## Abstract

*Introduction.* Systematic evaluation is an integral part of the organization and delivery of community oral health care programmes, ensuring the effectiveness of these community-based interventions. This study aimed to assess the knowledge and practice of primary health care (PHC) personnel regarding their duties toward oral health. *Methods and Material.* A cross-sectional study was carried out among three groups of PHC personnel in the city of Kerman (Iran). Volunteer personnel completed a piloted questionnaire which included demographic data, some question regarding their knowledge about oral health, their duties and also their practice regarding public oral health. All data were analyzed using chi-square and Pearson correlation test. *Results.* One hundred and fifty-seven out of 225 eligible personnel participated in the study. Sixty percent were auxiliary health workers (Behvarz). All personnel had a good level of knowledge regarding oral health. Despite significant differences among the knowledge of the personnel toward oral health, there was no significant difference between their knowledge related to their duties regarding oral health. The auxiliary health worker group had a higher rate (45.6%) for better public oral health practice. *Conclusion.* The study showed the personnel have good knowledge of their duties regarding oral health. However, their practice is not in line with their knowledge and needs more attention.

## 1. Introduction

Tooth decay and gum disease are among the most common diseases in human society, which take up a lot of family time and expenditure [1]. The national oral health survey which was carried out in 1997 showed that the average DMFT in children aged 12 in Iran was 1.5 [2]. About 80% of this value includes carious tooth and this figure rose to 1.8, according to the next national oral health survey which was carried out by the oral health bureau in the year 2003 [3]. Since then, the reasons behind the spread and occurrence of oral diseases were recognized and their prevention was emphasized. The reduction of these illnesses in many countries around the world is thanks to the prevention philosophy. The first step in prevention is to promote the culture of oral health in people [4].

After the WHO declaration in Alm-Ata in 1979 to try to achieve the goal of “Health for All” and the efforts for creating a primary health care (PHC) network around the world and in Iran, eventually dental and oral health services in a lot of countries were passed on to this network [5]. In turn, in 1994 oral health services in Iran were integrated with the PHC network. Expansion of this network across the country could be good, so oral health care could be accessible for all the members of the community [6].

In Iran, the health care service system has been established in such a way that, wherever people live, they can access basic health care services with consideration of geographical and population circumstances. A widespread PHC network in the country enabled this in the field of public care. But unfortunately oral health still has not completely found its place [6].

The plans for integrating oral health as part of the PHC network emphasizes at the first level the prevention services and at the second level the treatment services. According to the plan mentioned, some of health service personnels beside the dental care personnels are involved in oral health by giving out oral health care services. These health care personnels include [7]

1. Auxiliary Health Worker (AHW known as Behvarz), who is trained to give the first level of the preventive programme such as vaccination to children and, during pregnancy, care to women in rural health house,
2. Health Visitor (HV) who gives the preventive programme at urban or suburban health centers,
3. Health Technician (HT) who is an expert in family and child health care, giving consultation on most fields in public health.

These personnels are mainly responsible to give primary oral health care services to communities in partnership with dental professionals' personnel. A job description, alongside target groups which include pregnant women, children from birth to 6 years old, and children aged between 6 and 12, for the personnel has been stated [6].

The duties of the personnel in this job include educational services on oral health, screening for oral diseases, and also referring the target groups to dentist and specialists. These duties show the importance of these groups of personnel in promoting oral health which of course is only so if these personnels are aware of their responsibilities.

After creating the PHC system in many countries and integrating oral health services, especially prevention services in this system, different studies have been carried out to measure the effectiveness of these plans and the role of health personnel in promoting health [8, 9].

A method for evaluation of oral health programmes is to assess the practice of the health care personnel and its outcome on the public health.

In different countries such as Bangladesh, Indonesia, Nepal, Tanzania, and even some developed countries, research has been done concerning the PHC personnel, but these research efforts were based on general knowledge of the workers toward oral health, not on the specific job description [10, 11]. In one study in Iran regarding the level of knowledge of health workers in relation to oral health in the city of Yazd in Iran, it was shown that their knowledge was acceptable but lacking in some areas [12]. In another study, carried out in Qom on their health care personnel, a relationship was observed between the level of education and the level of knowledge possessed and also an inverse relationship between the years in service and the amount of information they had [13]. A different study carried out in Lahijan showed that the knowledge of school health instructors about oral health increased with the amount of years they were in practice [14].

Another study in the Yazd province showed that the level of information of health instructors in schools toward oral health was low [15]. However, no study has been carried out in relation to the job description of the personnel regarding oral health and the services given based on that in Iran.

Any research on recognizing the level of knowledge, kind of conception and practice of health carers that are busy in health care units in the network will certainly help in the way of scientific and specialized education of the workers. It will also help in the quality of the health services and the health education given. Therefore, the aim of this study was to show a picture of the situation in the city of Kerman which is in the largest province in Iran. Determining the amount of dental service given by these groups of PHC personnel might be used to improve the integrating plan in future. The main objective of the study was to determine the level of knowledge and practice of three groups of PHC personnel in Kerman and its outskirts regarding their job duties toward oral health.

## 2. Method and Material

 The study considered a cross-sectional analytical methods and 3 groups of health care personnel (as mentioned in Section 1) were under investigation.

The information regarding the number of health care centers which are actively working in the city and its outskirts and the number of each of their personnels was acquired from the Health Vice Chancellor of the Kerman Medical Sciences University.

Sampling was on the basis of a census of all eligible personnel; they were informed about the nature of the study and invited to participate.

According to the information released, there were 36 centers in Kerman and 20 centers in the outskirts, which altogether held 225 personnel.

Volunteer participants signed an informed consent form, after the required information was received, and reassurance was given that the information would be kept confidential. The study was approved by the research ethics committee of the Vice Chancellor of Research of Kerman Medical Sciences University.

Information was collected by completing a questionnaire which was piloted to insure the given information is genuine. The questionnaire had 3 parts:

1. questions regarding general knowledge of the personnel about oral health,
2. questions of personnel knowledge toward their duties regarding oral health listed in the integration programme,
3. questions regarding the practice of personnel toward oral health duties which are mentioned in the programme.

The questions on the level of knowledge about oral health were based on the common contents of the teaching curriculum of the target groups [16] and the duties listed in the PHC network and integration program [17]. The questions were multiple choice and true or false. Some demographic data, job history, and information about any educational courses in oral health were also collected.

To prevent interference in the questions related to the knowledge of listed duties and the questions about the practice, first the knowledge questions were handed out and after that the practice questions. To analyze the data, the knowledge questions were marked, right answers marked 1 and wrong answers marked zero and the “I do not know” option was also marked zero. Each person's score was calculated as sum of the gained marks. A score of 80% was considered as high (good) knowledge, 50%–80% meant moderate knowledge, and less than 50% was equal to low (weak) knowledge. Questions regarding the practice were categorized to good, medium, and weak practice, and the percentage of personnel in each category was calculated. Relations between different variables were tested using chi-square and Pearson correlation test. A *P* value of 0.05 was used throughout the analysis.

## 3. Result

Seventy percent of eligible personnel (157 out of 225) participated in the study, including 31 health technicians (19.7%), 34 health visitors (21.7%), and 92 auxiliary health workers (58.6%).

The mean age of the participant was 34.5 (±7.1) and their average working years 12.7 (±6.9) years (Table 1).

**Figure 1:**
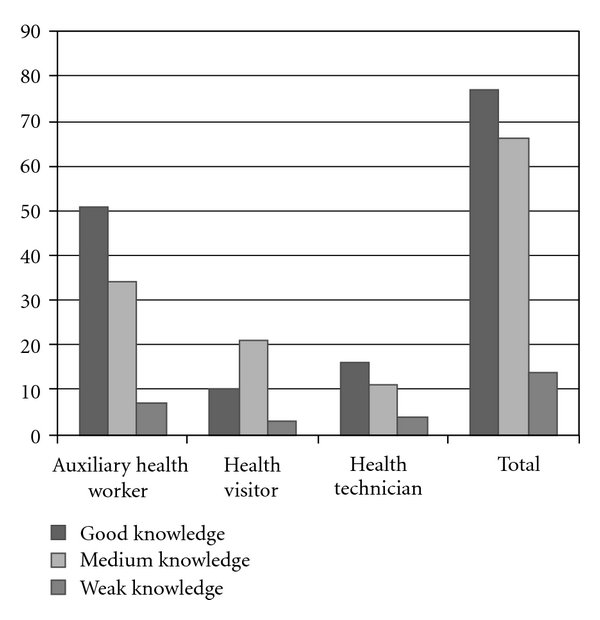
The knowledge level of participants about oral health for each group.

About 30% (46) were male and 70% (111) were female. The level of general knowledge in relation to oral health was as follows (according to Figure 1):

(1) $$\begin{array}{cc} & \text{Auxiliary}\;\text{health}\;\text{worker}\;\text{knowledge}\\ & \;\;>\text{health technician knowledge}\\ & \;\;>\text{health visitor knowledge}. \end{array}$$

There was a significant difference between the level of personnel general knowledge toward oral health (*χ* ^2^ = 8.24, *P* = 0.01) as shown in Table 2.

The level of knowledge of the three groups of the personnel about their duties in the field of oral health showed no significant difference (*χ* ^2^ = 3.78, *P* = 0.345) (Table 3).

Pearson's correlation test showed a positive but weak relation between the level of personnels general knowledge toward oral health and the level of their knowledge regarding their duties (*r* = 0.263, *P* = 0.01).

In relation to practice, frequency distribution of the participants' answers about obeying their duties relevant to oral health in this study has been shown in Table 4.

The results of how much the personnel in each of the three groups did their duty related jobs well, is as shown below:

(2) $$\begin{array}{cc} & \text{Auxiliary}\;\text{health}\;\text{worker}\;\left(45.6\text{\%}\right)\\ & \;\;>\text{health}\;\text{visitors}\;\left(26.5\text{\%}\right)\\ & \;\;>\text{health}\;\text{technicians}\;\left(12.9\text{\%}\right). \end{array}$$

A significant difference was observed between the quality of work of the three groups (*χ* ^2^ = 35.5, *P* = 0.00) as shown in Table 5.

A positive but weak correlation between level of knowledge of job description and practice in the three groups of was observed (*r* = 0.224, *P* = 0.05).

There was also a negligible relativity between level of general knowledge of oral health and how well the practice was done (*r* = 0.19, *P* = 0.816).

There was no significant difference between male and female personnels in relation to their knowledges (*χ* ^2^ = 0.532, *P* > 0.05) and practice (*χ* ^2^ = 4.64, *P* > 0.05) toward oral health duties.

Approximately 75% of the participants had no figure of oral health status (DMFT/CPITN indices) of those under their care.

Fifty percent of respondents did not have any training course on oral health, and therefore they assessed that their own information in this topic, was not enough (Table 6).

They also believed that more training program in oral hygiene and care could be beneficial. On the other hand, 40% of them thought it would be more specialized if dentists and oral hygienists were used for oral health care practice in public health.

## 4. Discussion

The reported study aimed to assess part of the integration program of oral health in the PHC network and was conducted on three groups of personnels involved in this program and was targeted at their responsibilities in relation to this program.

The level of knowledge of most of the personnels about oral health was generally of a good standard. There was a significant difference between the three groups mentioned (*χ* ^2^ = 8.22, *P* = 0.01). The auxiliary health worker group had a higher level of knowledge even though they had a lower level of education. These results were similar with those acquired in the study carried out on the level of knowledge this group had about oral health in Yazd, in which the questions were similar [12].

The difference in the knowledge level of these personnel can be for one of two reasons.

The first reason being the fact that Oral Health Bureau designed a book as a reference for the subject of oral health to train the auxiliary health worker group. This book's contents are new and have all the useful information needed in the field of oral health [16]. Secondly, due to the group's work conditions they are mainly located in areas where access to dental personnel is unavailable so they are faced with more questions and problems and are obliged to have more information in this field. Also the role of continuing professional education (CPE) programs arranged for this group should not be ignored.

 Although the majority of participants were women (70.7%), in comparison of the level of knowledge no significant difference was observed between male and female personnels (*χ* ^2^ = 0.532, *P* > 0.05). However in the research carried out by Pourhashemi on health care personnel of Qom, women had a higher knowledge compared to men [13]. Also in the study which Dastjerdi and colleagues carried out on school hygiene instructors in Yazd, men had higher knowledge than women [15].

Considering the thorough job description required of the personnel in oral health, the results show a moderate to-high-standard level of knowledge in near to 90% to these three groups of their duties. No considerable difference was seen between these groups (*χ* ^2^ = 3.782, *P* = 3.345).

In the research carried out by Taghavi and colleagues in Yazd in 2000, the lack of decent knowledge was shown in auxiliary health worker in some areas relating to the teaching of oral hygiene [12]. But the difference which was observed in this study and increased level of knowledge in auxiliary health workers in their duties show some improvements in organization or learning centers in recent years.

The results of the study showed a weak relation between the general knowledge of these three groups on oral health and their knowledge of their responsibilities (*r* = 0.263, *P* = 0.01). However this can be due to the contents of the job description which is not linked to the general knowledge in this area. Even if the individual, for example, is not aware of some of the topics linked to oral health, he/she can do what is required based on the forms filled out by the service receivers and target groups so as a result he would be familiar with the job description.

In regards to practice according to the job description, considering the results, which show that the auxiliary health worker group did the best by 45.6%, there is a meaningful difference between the three groups in this field (*P* = 0.00). This occurrence can be due to the fact that auxiliary health worker personnels are more involved considering their situation and their referrals and work conditions.

Also there was a weak relation between the function of personnel in these three groups and their knowledge about oral health which could be due to the fact that they are only doing their duties which are listed under their job description and could even be without the needed information related to oral health.

Evaluation of the work of these personnel in relation to oral health especially in an educational manner can be done by examining the teachings given in schools and nurseries which include the target group of children aged less than 12. Approximately 30% of the personnel responded that they embarked in carrying out these teaching sessions and 20% admitted they had never had a teaching session. As it was mentioned earlier, analyzing the oral health of 12-year olds in 1993 showed an average DMFT of 1.5. The report of the research carried out in 2003 by the office of oral and dental health reported the existence of a lot of dental decay in these age (0–12), in Iranian children [2, 3]. Considering that the oral health services have been integrated in the PHC network in 1994, this topic has yet got room for debate and thought and it seems that more thought should be given to this problem. However as there are numerous factors which contribute to tooth decay, discussing this matter should be done cautiously.

An interesting point which was highlighted by the personnel under study was that 75% of them were not aware of the oral health status of the people under their care. This can be as a result of oral health not being a priority in health centers or it could be results of lack of skills to collect the data linked to this matter.

When evaluating whether this job description and these personnel's duties on oral health of schoolchildren is useful or not, it was observed that the majority of opinions stated that their usefulness was dependant on further education of these personnels.

It seems it is a must that these personnels first need to be aware themselves of the importance of oral health and its link to general health and gain the skills and information required in this area, before they can act out their duties [18–21].

The integration of oral health services in the PHC network in different countries such as Nepal, Tanzania, Indonesia, and Thailand has been carried out, and evaluation of this programme showed the lack of its efficiency in primary oral health care which this research in Iran confirms these study results [10, 11].

As oral health still is not a high priority in many countries' population, the emphasis should be on prevention techniques, especially the common risk factor approach and using the PHC workers in this field might help also to improve oral health [22].

It could be pointed out, to reach the goals set by WHO in the oral health area by 2020, there is a choice to make better use of the PHC system considering it being widespread [23].

It is clear that until now no study on the precise practice specifications of the PHC personnel's duties in the dentistry area has been carried out in Iran. Although some limited studies have been carried out in Qom, Gorgan, and a few other cities on the educational methods of oral hygiene or the level of general knowledge which personnel has in relation to oral health [12–15]. Maybe the results of this study which were based on the work done in accordance to job description can be a major step in part of the integration plan and force policy makers to change the practical plans.

In this study there were some limitations in terms of accessibility to personnel and the lack of cooperation from many of them to participate in the study. This in turn caused a fewer of them to take part voluntarily. But the results show that even though the personnels have approximately enough information on oral health and were to some extent familiar with their duties in this area, they had an unsatisfactory performance. It is up to the people in charge to hold justification meetings or to convince the personnel of the importance of this subject either indirectly or directly through their specialists and offices. Therefore to make better use of the research's results, the statements below have been recommended.

1. Training of special work forces such as oral hygienists which specifically tend to educational and prevention matters linked to oral health. Having an independent force in the network for this purpose better displays the importance of oral health.
2. Carrying out local selection of forces—similar to auxiliary health worker forces—based on the different needs in different rural and urban areas.
3. Organizing education and CPE programs suitable for the personnel involved in the integration program until a sufficient number of oral and dental hygienists have been trained.
4. Organized observation and control over the work of the personnel involved in the integration program in regards to the job description related to them and rewarding or punishing them in accordance to this.
5. Providing required resources and equipment for the personnel to give oral health care to the population under their care.
6. Making a logic connection between dentistry and PHC units in relation to their job description and convincing the personnel and the dentists in such a way that dentists, as well as accepting their responsibility indicate the personnel as the operation arm of oral health care services.
7. Planning new educational books based on documents and strong scientific evidence to train personnel, and target group, and providing sufficient financial resources to carry out learning programs in this field.

## Figures and Tables

**Table 1:** The mean age and time in work for each job in years.

Job group	AHW* (*N* = 92)	HT* (*N* = 31)	HV* (*N* = 34)	Total (*N* = 157)
Age (year)	35.5 ± 5.6	35.2 ± 10	31.1 ± 7	34.5 ± 7.1
Time in work (year)	13.7 ± 0.6	15 ± 7.7	8.2 ± 6.2	12.7 ± 6.9

*Auxiliary Health Worker (AHW); Health Visitor (HV); Health Technician (HT).

**Table 2:** Comparison the level of general knowledge toward oral health among the study groups.

Job group (level of knowledge)	AHW	HT	HV	Total
Good	51 (55.4%)	16 (51.6%)	10 (29.4%)	77 (49%)
Medium	34 (36.9%)	11 (35.5%)	21 (61.8%)	66 (42%)
Weak	7 (7.7%)	4 (8.8%)	3 (8.8%)	16 (9%)
Total	92 (100%)	31 (100%)	34 (100%)	157 (100%)

(*χ* ^2^ = 8.224, *P* = 0.01 sig).

**Table 3:** Comparison the level of general knowledge of participants toward their duties related to oral health in each job.

Job group (Level of knowledge toward their duties)	AHW	HT	HV	Total
Good	66 (71.7%)	18 (58%)	20 (58.8)	104 (66.2%)
Medium	22 (24%)	10 (32.3)	11 (32.4)	43 (27.4%0
weak	4 (4.3%)	3 (9.7%)	3 (8.8%)	10 (6.4%)
Total	92 (100%)	31 (100%)	34 (100%)	157 (100%)

(*χ* ^2^ = 3.782, *P* = 0.345 N sig).

**Table 4:** Frequency distribution of answers relating to the questions toward practice relevant to the job description of oral health.

Frequency (%) questions	Most often	sometimes	Not often	never	No answer
How often are you giving oral health education to school (nurseries) children?	48 (19.7)	70 (44.6)	19 (12.1)	20 (12.7)	—
How do you supervise fluoride mouth wash programme in school?	31 (19.7)	54 (34.4)	20 (12.7)	52 (33.2)	10 (6)
Are you examining mouth and teeth of pregnant women?	59 (37.6)	35 (22.3)	17 (10.8)	46 (29.3)	—
How do you refer the patients to dentist or oral hygienist?	76 (48.4)	37 (23.6)	19 (12.1)	25 (15.9)	—
	yes	no	No answer		
Have you had any partnership with parents regarding oral health?	45 (28.7)	112 (71.3)	—		
Have you had any partnership with other health sector regarding oral health?	24 (15.3)	133 (84.7)	—		
Do you know about the water fluoride content of your area?	26 (16.6)	107 (68.1)	24 (15.3)		
Do you have any information about oral health status (DMFT/CPITN) of the population under your care?	55 (35)	102 (65)	—		
Do you complete the annual oral health report form?	76 (30.2)	81 (51.6)	—		

**Table 5:** Comparison of the practice by participants in the study in relation to their duties listed in oral health field.

Job group (Practice toward their duties)	AHW	HT	HV	Total
Good	42 (45.6%)	4 (12.9%)	9 (26.5%)	55 (35%)
Medium	42 (45.6%)	13 (41.9%)	19 (55.9%)	74 (47%)
Weak	8 (8.8%)	14 (45.2%)	6 (17.6%)	28 (18%)
Total	92 (100%)	31 (100%)	34 (100%)	157 (100%)

(*χ* ^2^ = 35.57, *P* = 0.00 sig).

**Table 6:** Frequency distribution of the answers given by the participants about whether or not they should go on learning courses and how they assessed their own information.

Question	Answer	Frequency (%)
Have you had any training course about oral health?	Yes	31 (19.7)
	No	33 (21)
	No answer	93 (59.20)

How do you assess your knowledge about oral health?	Enough	24 (28.6)
	Low	45 (53.6)
	High	15 (17.8)
	No answer	73 (46.4)
